# What Impacts Early Language Skills? Effects of Social Disparities and Different Process Characteristics of the Home Learning Environment in the First 2 Years

**DOI:** 10.3389/fpsyg.2020.557751

**Published:** 2020-12-08

**Authors:** Manja Attig, Sabine Weinert

**Affiliations:** ^1^Competencies, Personality, Learning Environments, Leibniz Institute for Educational Trajectories, Bamberg, Germany; ^2^Department Psychology I – Developmental Psychology, University of Bamberg, Bamberg, Germany

**Keywords:** vocabulary, grammar skills, home learning environment (HLE), social disparities, quality of interaction behavior, picture book reading, first 2 years

## Abstract

It is well documented that the language skills of preschool children differ substantially and that these differences are highly predictive of their later academic success and achievements. Especially in the early phases of children’s lives, the importance of different structural and process characteristics of the home learning environment (HLE) has been emphasized and research results have documented that process characteristics such as the quality of parental interaction behavior and the frequency of joint activities vary according to the socio-economic status (SES) of the family. Further, both structural and process characteristics are associated with children’s language development. As most of the studies focus on single indicators or didn’t take the dynamics of parenting behavior across age into account, the present paper aims to investigate the associations of different characteristics of the home learning environment as well as their potentially changing impact on the language skills of 2-year-old children. Using data of 2.272 families of the infant cohort study of the German National Educational Panel Study (NEPS), longitudinally assessed process characteristics (sensitivity in the sense of maternal responsivity to the child’s behavior and signals in mother–child interaction; maternal stimulation behavior which goes beyond the child’s actual level of action and development; frequency of joint picture book reading) and structural characteristics (mother’s education, equivalised household income, parental occupational status) were considered. Language skills (vocabulary and grammar) of the children at the age of two were measured by a standardized and validated parent report instrument (child language checklist). Results showed that (1) all three process characteristics of the home learning environment (HLE) are associated with the family’s SES; (2) across three assessment waves nearly all process characteristics predicted children’s vocabulary and grammar skills with some process-specific changes across waves; (3) despite separate direct effects of nearly all HLE-process characteristics in each wave, the amount of explained variance in a joint model including the HLE facets from each wave is hardly higher than in the separate models; and (4) socioeconomic background predicted both language facets of the children in each model even when controlling for the assessed process characteristics of the home learning environment.

## Introduction

From the very beginning children differ in their individual resources, basic abilities and other characteristics as well as in their developmental progress. Bioecological models of development (e.g., Bronfenbrenner and Morris, 2006) state that from early on – besides individual prerequisites – the learning environments impact the development of children. Accordingly, plethora of research has documented a significant association between different kinds of learning environments and child development (e.g., Hart and Risley, 1995; Nord et al., 2000; Anders et al., 2012; Weinert and Ebert, 2013; Lehrl et al., 2020). Focusing on the first years of life, the family is seen as the most important learning environment (Bornstein, 2002) and parenting behavior has been emphasized as particularly important for the development of children (e.g., NICHD Early Child Care Research Network, 2002a,b). Further, research showed that the early home learning environment also predicts the quality of the later home learning environment (Toth et al., 2020). According to the educational framework of the home learning environment (e.g., Kluczniok et al., 2013), structural characteristics such as parental education, occupation, and household income [as indicators of the socioeconomic status (SES) of the family] affect educational processes (e.g., quality of interaction behavior, joint activities). These educational processes in turn impact child development. In line with these assumptions, research has shown that structural characteristics (SES) as well as process characteristics of the home learning environment (e.g., the above mentioned quality of interaction behavior) are associated with cognitive, socio-emotional, and especially language development in childhood (e.g., Hart and Risley, 1995; NICHD Early Child Care Research Network, 1998, 2002a,b; Kluczniok et al., 2013; Weinert and Ebert, 2013; Bradbury et al., 2015; Friedman-Krauss et al., 2016; Hurt and Betancourt, 2016). Overall and in line with the educational model outlined above, research findings suggest that SES-related disparities in language development could be traced back to differences in process characteristics of the home learning environment which are associated with the families’ socioeconomic status; however, only few studies address the dynamics of parenting behavior across the first years (Lugo-Gil and Tamis-LeMonda, 2008; Rodriguez and Tamis-LeMonda, 2011; Vallotton et al., 2017). Hence, one aim of the present study is to take a longitudinal perspective by investigating the effect of social background on different characteristics of educational processes, particularly the interaction behavior of the mother (with a focus on maternal sensitivity and stimulation behavior) as well as joint activities across the first 2 years.

Language development is seen as a key factor for later development as well as for school readiness, reading skills, and school success (NICHD Early Child Care Research Network, 2005a; Rowe et al., 2012; Schuth et al., 2017; Weinert and Ebert, 2017; Rose et al., 2018). However, language is not a unitary phenomenon and is comprised of various components or subdomains, including vocabulary and grammar. As these components have been suggested to be differentially related to variations in the early home learning environment (Vasilyeva and Waterfall, 2011) and as language proficiency mutually draws on both components, our study addresses both, early vocabulary and early grammar. In particular, vocabulary is often seen as an indicator of language as well as of more general knowledge acquisition and crystallized intelligence and is thus highly prone to environmental stimulation (e.g., Kail and Pellegrino, 1985; Hart and Risley, 1995; Hoff-Ginsberg, 1998; Weinert et al., 2007; Rowe and Goldin-Meadow, 2009). Early grammar, however, is sometimes conceptualized as a more “inside-out” developmental phenomenon (see e.g., Golinkoff and Hirsh-Pasek, 1990; Hirsh-Pasek and Golinkoff, 1996) with environmental input not being the driving force, but instead an enabling force particularly in the early phases of development (Newport et al., 1977; Huttenlocher et al., 2010). Thus, the second aim of our study is to investigate how structural (SES) and the longitudinally assessed process characteristics of the early home learning environment (HLE) predict each language facet at the age of 2 years when the home learning environment plays a major role and whether the association with different environmental processes of the HLE changes over time and varies according to the language component considered.

## Theoretical and Empirical Background

### SES-Related Disparities in Process Characteristics of the Home Learning Environment

As already mentioned, SES-related differences in child development are suggested to be mainly transferred by qualitative and quantitative differences in the home learning environment (e.g., Lugo-Gil and Tamis-LeMonda, 2008). Educational frameworks (e.g., Kluczniok et al., 2013) assume that structural characteristics of the family (SES) affect process characteristics and hence the quality of the home learning environment (e.g., Anders et al., 2012; Lehrl et al., 2012; Weinert et al., 2017). In particular, family stress and family investment models (Haveman and Wolfe, 1994; Conger and Donnellan, 2007) presume that higher education, lower economic hardship, and more social resources (e.g., higher occupational status) impact on materials (e.g., books at home), living conditions, and family processes as these parents may, among other factors, gather more information on child development, experience less parental distress, and may thus be more able to provide their children with a high quality home learning environment.

A large number of research findings are in accordance with these assumptions. For example, the overall quality of the home learning environment, as measured by the Home Observation for Measurement of the Environment Inventory (HOME, Caldwell and Bradley, 1984), as well as the quality of parent–child interactions proved to be associated with the education level of parents (Bradley et al., 2001; Lugo-Gil and Tamis-LeMonda, 2008; Rowe, 2008; Magnuson et al., 2009; Neuhauser, 2018). Further, findings also showed that poverty is associated with a decreased quality of interaction behavior and a decreased quantity and quality of language input (Hart and Risley, 1995; Hoff, 2003; NICHD Early Child Care Research Network, 2005b; Rowe, 2008; Gudmundson, 2012).

With respect to SES, various aspects of the quality of interaction behavior have been documented to be associated with social disparities (Bradley et al., 2001; Gudmundson, 2012; Weinert et al., 2017; Attig and Weinert, 2018). Using data of the German National Educational Panel Study (NEPS), Weinert et al. (2017) found that maternal education predicted how sensitive and stimulating the mother interacted with her 7 months old child. This converges with research results demonstrating an association between SES and parent’s sensitivity at 12 months of age (Bernier et al., 2010) as well as with findings from the NICHD study (NICHD Early Child Care Research Network, 1999). Thus, mothers with low levels of education and mothers in low income households have been shown to provide their children with a less sensitive and less stimulating home learning environment (Klebanov et al., 1994; Bradley et al., 2001; Dilworth-Bart et al., 2007; Gudmundson, 2012; Neuhauser, 2018). In addition, focusing on language stimulation behavior, findings showed that – for example – mothers with a high socio-economic status talked more to their children compared to lower SES mothers (Hoff et al., 2002). Further, differences according to the family’s SES were not only found for spontaneous speech and verbal communication but also with respect to the frequency and quality of joint book reading (Bradley et al., 2001; Niklas and Schneider, 2010; Farrant and Zubrick, 2012; Lehrl et al., 2012; Hayes and Berthelsen, 2020).

To sum up, SES-related differences have been documented for different facets of parenting behavior and the quality of parent–child interaction. As these facets are highly important for the development of children it is assumed that these early differences in parenting behavior in turn affect child development. Restrictions in qualitative and quantitative facets of the home learning environment are seen as a risk factor for child development. Thus, analyzing the impact of social background on facets of parenting behavior longitudinally in the first years of life will help to better understand the specific and potentially changing role of different facets of the home learning environment.

### Disparities in Children’s Language

Multiple studies have shown SES-related differences in various areas of child development such as socio-emotional, cognitive, and language development (e.g., Hart and Risley, 1995; NICHD Early Child Care Research Network, 1998; Weinert and Ebert, 2013; Bradbury et al., 2015; Friedman-Krauss et al., 2016; Hurt and Betancourt, 2016). For instance, Hart and Risley (1995) documented that children living in low-income households have smaller vocabularies and more restricted language skills compared to their more advantaged peers. Further, they also showed SES-related differences in the verbal learning environment of the children (the so-called “30 million word gap”) which is also shown to be associated with the development of children (Hart and Risley, 1995). In contrast, a recent study by Sperry et al. (2018) showed only weak SES-related disparities in the number of words children heard (see Golinkoff et al., 2019; Sperry et al., 2019 for a critical discussion of the findings). Yet, Nord et al. (2000) had documented that children living in poverty or children with two or more educational risk factors were less likely to recognize the letters of the alphabet, count to 20 and higher, write their names, or read or pretend to read a storybook compared to their more advantaged peers. With respect to different aspects and indicators of language development, findings revealed that children from low-SES homes showed lower levels of oral language skills compared to children from more advantaged backgrounds (Weinert and Ebert, 2013; Linberg and Wenz, 2017; Law et al., 2019). This holds true for language processing skills, language comprehension as well as language production at different ages (Hoff, 2006; Fernald et al., 2013).

These results on the association between economic strains and children’s language skills converge with studies focusing on disparities according to maternal education. Thus, maternal education is shown to be associated with receptive and expressive language skills of 4-year-old children (Reilly et al., 2010), the language performance of 5-year-olds, as well as with the longitudinally assessed language performance of 3, 4, and 5 year olds (Weinert and Ebert, 2013).

Further, SES-related disparities in language skills are not only found in preschool or school age children (e.g., Law et al., 2012; Linberg and Wenz, 2017) but are also evident in even younger children below the age of 3 years (Halle et al., 2009; Fernald et al., 2013; Attig and Weinert, 2019; Law et al., 2019). For example, interrelations between parental education as well as occupation with vocabulary were already shown in 18-month-old children (Fernald et al., 2013); and at the age of 24 months, a 6-month gap in language processing skills was evident (Fernald et al., 2013). Looking at 2-year-old children, Law et al. (2019) as well as Attig and Weinert (2019) showed that structural characteristics as well as process characteristics in the second year of life were likely to affect the toddlers’ language skills.

A lot of research focuses on vocabulary size and it appears that it is the aspect of language which is most sensitive to vary according to SES (Rescorla, 1989; Hart and Risley, 1995; Arriaga et al., 1998; Hoff-Ginsberg, 1998; Dollaghan et al., 1999; Hoff, 2003; Pan et al., 2005; Rowe and Goldin-Meadow, 2009). In contrast, early grammar skills - in accordance with the so-called nativist theories of language acquisition (e.g., Fodor, 1983; Pinker, 1984; Chomsky, 1988; Van der Lely and Pinker, 2014) – were argued to be less influenced by the home learning environment (Vasilyeva and Waterfall, 2011). Although the empirical findings concerning grammar development seem to be somewhat inconsistent and controversial, there is a growing amount of research showing – in accordance with the more social-cognitive theories of language acquisition (Elman et al., 1996; Tomasello, 2003; Karmiloff-Smith, 2015; Weinert and Grimm, 2018) – that not only vocabulary but also child grammar varies according to SES and SES-related differences in the home learning environment (e.g., Vasilyeva et al., 2008; Huttenlocher et al., 2010; Weinert and Ebert, 2013, 2017; Anderka, 2018). For example, Weinert and Ebert (2013) showed SES-related differences in the receptive vocabulary as well as in the receptive grammar of 3-year old children which remained stable across preschool age. This result converges with findings from other studies which also found that children from high SES families outperform lower SES children on language tests including measures of grammatical development (Morisset et al., 1990; Dollaghan et al., 1999) and on various measures of productive and receptive syntax (Huttenlocher et al., 2002). However, it has been presumed that the early stages of grammar acquisition, below age three, may be less prone to environmental variation and more determined by innate factors (Anderka, 2018 for a brief overview; see also the results of Huttenlocher et al., 2010). Thus, from a theoretical as well as from an empirical perspective it seems worthwhile to differentiate both language components and to not only consider vocabulary but also early child grammar when investigating effects of the home learning environment on early child language as SES-related educational processes might affect them differentially.

### The Impact of Process Characteristics of the Early Home Learning Environment on Language Skills

As already mentioned, different facets of the home learning environment are associated with child development (e.g., Melhuish et al., 2001, 2008; Lugo-Gil and Tamis-LeMonda, 2008; Melhuish, 2010). For instance, focusing on language development, the NICHD study revealed a significant relation between maternal sensitivity and child vocabulary at the age of three (NICHD Early Child Care Research Network, 1998). In addition, research results showed maternal sensitivity to be associated with speech comprehension and various milestones of language development (e.g., Ruddy and Bornstein, 1982; Tamis-LeMonda et al., 2001; Paavola et al., 2005; Nozadi et al., 2013). Thus, for example, the children of more sensitive mothers began to talk earlier and reached the milestone of a 50-word vocabulary at a younger age than children of less responsive mothers (Tamis-LeMonda et al., 1996; Tamis-LeMonda et al., 1998). In addition, maternal sensitivity at the age of 18 months predicted later language skills (Nozadi et al., 2013).

Most of the above-mentioned studies focused on maternal sensitivity or on a composite score of various facets of parental sensitivity and supportive behavior when investigating the association between interaction quality and language skills of children (e.g., NICHD Early Child Care Research Network, 2002a,b). Drawing on attachment theory (e.g., Ainsworth and Bell, 1970), parental sensitivity or responsivity is defined as a prompt, contingent, and suitable reply to the infant’s signals and needs (Ainsworth et al., 1974; Tamis-LeMonda et al., 2014). Such parenting behavior has been suggested to be highly relevant to child development as it allows the child to experience him- or herself as competent and valued and to explore the environment from a secure base (Bowlby, 1988). As a second facet – in accordance with Vygotsky’s theory and the concept of the zone of proximal development (Vygotsky, 1978) – parental stimulation of child behavior (also called scaffolding, e.g., Bruner, 1978) has been demonstrated to foster child development; this facet of parenting behavior also includes a sensitive component as the parents have to read the child’s signals and to adapt their behavior to the child’s needs. Yet, stimulating parenting behavior goes beyond the child’s actual level of development or action thereby stimulating developmental progress by supporting the child in exploring the environment, by presenting the child with materials and language that amplifies the actual level of the child’s performance and offers new perspectives or exploration opportunities to the child. Recently Linberg (2018) has empirically demonstrated that it is useful to separate these two components even in the very first year of life.

In fact, not only the prompt, contingent, and adequate reaction of the mother to the child’s signals has been shown to be associated with language development, but also the described cognitively stimulating behavior that supports the child in exploring the environment and by presenting stimulating materials and toys to foster child development (Olson et al., 1986). For example, research findings showed that besides maternal responsivity at the age of 13 months, maternal verbal stimulation at the age of 24 months was associated with the children’s vocabulary progress (Olson et al., 1986). Vallotton et al. (2017) stated that different developmental periods of language development require certain parental behavior (see also Landry et al., 2001). In particular, they showed that maternal sensitivity at the age of 14 months had a stronger effect on vocabulary than cognitive stimulation. At the age of 24 months, both effects were small, but nearly the same size. At the age of 36 months, cognitive stimulation showed a stronger effect on vocabulary than sensitivity (Vallotton et al., 2017). Hence, whereas the effect of sensitivity on the vocabulary development of the children seems to be relatively consistent over the very first years, the effect of stimulation seems to grow throughout toddlerhood. These results fit well with the findings by Farah et al. (2008) who showed that stimulation, but not sensitivity, predicted children’s language skills at the age of 4 and 8 years.

However, different facets of mother–child interaction and the home learning environment might be associated with different areas of language development. For instance, Lehrl et al. (2012) showed that the quality of parent–child interaction predicted vocabulary but not grammar development, and the amount of experiences with books as well as the amount of complex language input (Anderka, 2018) predicted the development of receptive grammar but not vocabulary. Interestingly, SES-related disparities in vocabulary and grammar were also mediated by the respective factors (Anderka, 2018). Again, such results hint at the necessity to differentiate between language components as well as between different facets of parenting behavior which lead to a high quality of interaction behavior and home learning environment.

Hence, the present study focuses on sensitivity (in the sense of sensitive responsiveness) as well as on stimulation behavior in parent–child interaction as separate dimensions. Although studies such as the NICHD study (NICHD Early Child Care Research Network, 2002b; Belsky et al., 2007) longitudinally assessed the quality of interaction behavior, most analyses included composite scores. As parents adapt their interaction behavior to the behavior and the developmental status of their child (Rodriguez and Tamis-LeMonda, 2011) and because the effectiveness of features of the home learning environment may change over development (Olson et al., 1986; Vallotton et al., 2017; Korucu and Schmitt, 2020), it seems valuable to consider a longitudinal perspective on facets of parenting behavior in mother–child interaction across the first years to investigate possibly changing effects of the different aspects.

Not least and as already mentioned, not only the quality of interaction behavior has been shown to play a role in language development but also joint activities and the home literacy environment (e.g., Bus et al., 1995; Sénéchal and LeFevre, 2002; Farrant and Zubrick, 2012). A positive association with the frequency of joint picture book reading was shown – for example – for the vocabulary of preschoolers (Bus et al., 1995). Adding to this research, joint picture book reading explained variance in children’s expressive vocabulary and morphological knowledge in 4-year-old children (Sénéchal et al., 2008). Such results were not only documented for preschool or school-aged children. For example, Bromley (2009) showed that reading to the children at the age of 10 months is associated with their language skills at the age of 34 months (see also Rodriguez et al., 2005; Raikes et al., 2006 for findings in a similar direction).

In summary, different process characteristics of the home learning environment have been shown to predict later child language. Yet, most studies focused on only one or two aspects of the home learning environment. Possible interrelation between the facets of the home learning environment and their consequences for the language development of children were hardly considered (see for an exception Attig and Weinert, 2019; Law et al., 2019). Extending previous work (Attig and Weinert, 2019) which focused on three process characteristics, namely maternal sensitivity (as indicated by responsivity), mother’s cognitively stimulating behavior, and the frequency of joint picture book reading, as well as on structural characteristics, the present paper included longitudinal assessments of the process measures of the HLE allowing an investigation of the changes in the associations with SES across 2 years and across child outcomes as well.

## Present Study

Research indisputably shows significant associations between SES, parenting behavior, and child language with the home learning environment being a multidimensional construct. The present study considers structural as well as process characteristics of the home learning environment and adds to previous research by taking a longitudinal perspective on process characteristics of the HLE across the first 2 years. Thereby, the present paper extends previous research by Attig and Weinert (2019) which also considered three process characteristics as well as structural characteristics at one measurement point and analyzed their effect on the language skills of 2-years-olds. Attig and Weinert (2019) showed that maternal education as well as maternal sensitivity and stimulation behavior in mother–child interaction and, not least, the frequency of early picture book reading predict children’s language skills as indicated by a combined measure of vocabulary and grammar at age two. Using the same dataset [the newborn cohort study of the German National Educational Panel Study (NEPS), Blossfeld et al., 2011; Blossfeld and Roßbach, 2019]^1^, the present paper aims to address the following research issues and questions:

(1) Association of structural (SES) and various process characteristics of the home learning environment across the first 2 years of children’s lives.
  (a) Extension of findings on SES-related disparities in different process characteristics of the home learning environment across three measurement points;
  (b) Investigating the potentially changing associations between SES and various process characteristics of the home learning environment across three measurement points;

(2) Analyzing the predictive effect of structural (SES) and process characteristics on the early vocabulary and grammar outcomes of children at age two; in particular:
  (a) To what extent does the SES as well as different process characteristics in the first 2 years predict the vocabulary size of 2-year-old children and does the prediction differ when focusing on different time points in the first years of life?
  (b) To what extent does the SES as well as different process characteristics in the first 2 years predict early child grammar at 2 years of age and does the prediction differ across time points in the first years of life?

When considering SES effects on child development, studies differ according to which structural aspects, such as education, occupation, or income, they take into account. Some studies used single predictors (e.g., Reilly et al., 2007; Law et al., 2012) while others included a combination of different aspects (e.g., Weinert and Ebert, 2013). Overall, across studies the findings substantiate the assumption that – relatively independent of the SES-measure used – the association between SES and early language development seems to be robust (see also Hoff, 2013). In this paper we decided to not only focus on one aspect of the social background but to take the different facets of structural characteristics conjointly into account.

By differentiating the language components we also contribute to the issue of whether early child grammar is less influenced by environmental conditions compared to vocabulary in the early phases of child development as suggested by nativist accounts of language acquisition (e.g., Fodor, 1983; Pinker, 1984; Chomsky, 1988; Van der Lely and Pinker, 2014).

## Materials and Methods

### Sample

The present paper used data from the first three waves of the Newborn Cohort Study of the NEPS (Blossfeld and Roßbach, 2019). This cohort study includes a representatively drawn sample of around 3.500 children born between February and June 2012 and their families (Weinert et al., 2016). In each wave, a computer assisted parent interview as well as – amongst others – a parent–child interaction was conducted. In the first wave, the infants were 7 months old; in the second wave they were around 14 months when the parent interview was conducted and 17 months at the assessment of parent–child interaction. By design, only half of the sample (random selection) took part in the parent–child interaction during this wave. In the third wave, the children were 26 months old. For the present paper we included 2.272 families who provided data on the children’s language skills in the majority language (German) as an early outcome measure at 26 months of age. All families were excluded who reported only another language than the majority language (German) as interaction language at home. Hence, families with more than one interaction language are part of the analyzed sample as long as one of the interaction languages is German (see Table 1 for relevant descriptives on sample characteristics of the families and children included in the present study).

**TABLE 1:** Descriptives.

	**Mean/%**	**Median**	**Standard deviation**	**Minimum**	**Maximum**	***N***	**Missing**
**Maternal interaction behavior (5-point scales)^1^**							
Sensitivity w1	4,15	4,00	0,74	1	5	1.613	659
Sensitivity w2	3,44	3,00	0,71	1	5	926	1346^a^
Sensitivity w3	3,73	4,00	0,79	1	5	1.756	516
Stimulation w1	2,74	3,00	0,92	1	5	1.613	659
Stimulation w2	3,16	3,00	0,77	1	5	926	1346^a^
Stimulation w3	3,23	3,00	0,78	1	5	1.756	516
**Frequency of joint picture book reading**							
w1 (5-point scale)	3,05	3,00	1,46	1	5	2.272	0
w2 (5-point scale)	4,05	4,00	1,06	1	5	2.112	160
w3 (8-point scale)	7,44	8,00	0,93	1	8	2.272	0
**Child language (ELFRA)**							
Vocabulary	142,31		65,17	0	260	2.272	0
Grammar (standardized)	0		0,96	−2,25	1,82	2.058	214
**Socio-economic background**							
Education (low – high)		2,00	0,65	1	3	2.270	2
Income (Euro)	1.732,76		895,24	185,76	1.4285,71	1.925	347
HISEI	63,78		19,37	12,01	88,96	2.224	48
**Controls**							
Age (w3; in days)	805,79	802,00	32,066	676	977	2.271	1
Sex: girls	49%			0	1	2.272	0
Interaction language: German and other	22%			0	1	2.272	0

**^1^Levels of the interactional variables are not directly comparable across waves as the codings are adjusted to the age of the children (see Linberg A. et al., 2019). ^*a*^Missing by design. Education, maternal education. Income, equivalised household income (in Euro). HISEI, highest International Socio-Economic Index of Occupational Status. w, wave*.*

### Research Instruments

#### Home Learning Environment (HLE) – Process Characteristics

##### Parental interaction behavior

Adapted from the NICHD SECCYD study (NICHD Early Child Care Research Network, 1991), a semi-standardized interaction situation between child and mother^2^ was conducted at the families’ home during each of the first three assessment waves (Linberg A. et al., 2019; full sample in wave 1 and 3; half sample in wave 2). Parents were asked to play as naturally as possible with their child and the standardized toy sets. Interactions were videotaped and lasted 5 min in the first wave and 10 min in the second as well as in the third wave. Videos were coded afterward by extensively trained raters using qualitatively defined 5-point Likert scales (rating scales from 1 = *not at all characteristic* to 5 = *very characteristic*; adapted from NICHD Early Child Care Research Network, 1991; see Linberg A. et al., 2019 for a description). In the following analyses, we used the scales *sensitivity*, which focuses on the prompt and adequate reaction of the mother to the signals of the child, and (global) *stimulation*, which addresses the mother’s stimulating behavior (i.e., stimulation of speech and play). Inter-rater agreement was high (wave 1: 90% and 94%; wave 2: 92% and 95%; wave 3: 94% and 93%; Linberg A. et al., 2019).

##### Joint picture book reading

As another indicator of the home learning environment we considered the frequency of joint picture book reading in each of the three waves. Parents were asked on a 5- (first and second wave: ranging from not at all to several times a day) and 8-point-likert scale (third wave: ranging from never to several times a day) how often they or someone else in their home jointly engage in picture book reading with the child.

#### Child Language

To assess the children’s language skills at the age of two (wave three), the ELFRA-2 (Grimm and Doil, 2006) was administered. The ELFRA-2 is a standardized parental report measure on child language comparable to the internationally well-known “MacArthur-Bates Communicative Development Inventories (Toddler Form) – CDI” (Fenson et al., 1993). It includes a German vocabulary check-list of 260 words and phrases the child uses actively as well as 26 items on the child’s syntax and 11 items on morphological aspects, i.e., on the grammatical structures the child uses. The ELFRA is widely used and validated with scores correlating significantly with language test scores (for the validity of the ELFRA see Sachse et al., 2007). We used the vocabulary scale as well as an indicator of child grammar (mean of the standardized scales on syntax and morphology; inter-correlation *r* = 0,86).

#### Socio-Economic and Educational Characteristics of the Family

As structural aspects (SES), the following three variables, all measured in wave 1, were considered: first, the education of the mother based on the CASMIN classification (König et al., 1988) was used. The CASMIN classification was recoded into three categories (see Linberg T. et al., 2019 for a similar procedure):

– 1 = Low education (no qualification to intermediate secondary education without vocational qualification).
– 2 = Medium education (intermediate secondary education and higher education).
– 3 = High education (lower and higher tertiary education).

Second, we used the equivalised household income (in Euro) as an SES indicator; hence, the household income was weighted according to the persons living in the household (Organisation for Economic Co-operation and Development [OECD], 2013). In addition, the income was log transformed for structural equation modeling to reduce the skewness of this variable and to reduce a possible bias of increasing income in the high-income groups.

Third, the highest International Socio-Economic Index of Occupational Status (HISEI-08, Ganzeboom et al., 1992) of the family was included. The ISEI codes the prestige of the last occupation of a person. We used the highest ISEI of the parents.

#### Control Variables

When analyzing the language skills of the children, we considered the age of the child at wave three as well as the sex of the child as control variables as both are typically associated with early language development. Further, we considered the interaction language in the household (only German vs. children learning another language in addition to German; see also section on “Analytic Strategy” below).

### Analytic Strategy

A two-step approach was used. First, to investigate the effect of the social background variables on HLE process characteristics and, due to missing data, we used mixed-effects linear regression models. The models included social background as a fixed effect as well as a random intercept to account for the correlation between the repeated measures of the process characteristics. The mixed models were conducted in Stata 16. We used multiple imputation by chained equations (MICE) to handle missing values. Note that missing values were partially due to the design with a random selection of half of the sample conducting the interaction situation (see Table 1 for the amount of missing data for each variable). The imputation model included all three HLE process characteristics from each wave as well as education, equivalised household income, HISEI, and the different language measures. Further, to improve the imputation model, we added family status, psychological stress, and age of the mother as well as child’s negative affectivity to the imputation model. We created *m* = 50 imputed data sets using Stata 16. For the mixed models, all three assessments of HLE process characteristics were standardized as well as the three social background variables which were then averaged to create a combined SES measure. Three separate mixed models for each of the three HLE process characteristics were calculated.

In a second step, structural equation modeling (SEM) was applied to investigate the effect of the more distal (SES) measure and the more proximal process characteristics of HLE (maternal interaction behavior, joint book reading) on child vocabulary and child grammar at the age of two in separate models. The socioeconomic status (SES) of the family was modeled as a latent variable (see Weinert and Ebert, 2013 for a similar approach). All other variables were included as manifest variables. SEMs were calculated using M*plus* 8.2 (Muthén and Muthén, 2017) and Full Information Maximum Likelihood Estimation (FIML) was used to handle missing data in the predictors. With respect to control variables, we considered children’s age at wave three, the sex of the child, and the interaction language in the household (as these are typically associated with early language development) and regressed them on child language. The models allowed correlations between the HLE process variables and children’s sex and interaction language in the household. We started by analyzing the predictive effects for each wave separately and then combined all waves into one joint model to analyze the stabilities and the separate and joint impact of the predictors on child language across the first 2 years of the children’s lives.

## Results

Table 1 shows the descriptive characteristics of the analyzed variables across waves. Further, for all variables the amount of missing values is listed.

Turning to the correlations between the variables of interest, we found only low associations between the measures of maternal sensitivity over the three waves and low to moderate stabilities for mother’s stimulating behavior as well as the frequency of joint picture book reading (see Table 2 for all *r*’s). Correlations between the family’s SES and the three HLE process characteristics as well as with the two measures of child language were low but significant. Further, the correlations between the two language measures and the three HLE process characteristics were mostly low (see Table 2).

**TABLE 2:** Correlations.

	**1**	**2**	**3**	**4**	**5**	**6**	**7**	**8**	**9**	**10**	**11**
(1) Sensitivity w1	1										
(2) Sensitivity w2	0,16***	1									
(3) Sensitivity w3	0,15***	0,25***	1								
(4) Stimulation w1	0,27***	0,14***	0,06**	1							
(5) Stimulation w2	0,18***	0,34***	0,14***	0,34***	1						
(6) Stimulation w3	0,21***	0,16***	0,25***	0,22***	0,30***	1					
(7) Picture book w1	0,08***	0,13***	0,05*	0,17***	0,12***	0,07**	1				
(8) Picture book w2	0,14***	0,13***	0,11***	0,13***	0,18***	0,12***	0,34***	1			
(9) Picture book w3	0,16***	0,19***	0,16***	0,12***	0,13***	0,15***	0,23***	0,33***	1		
(10) Child vocabulary	0,16***	0,21***	0,20***	0,05*	0,20***	0,22***	0,19***	0,23***	0,27***	1	
(11) Child grammar	0,16***	0,20***	0,22***	0,04	0,18***	0,22***	0,18***	0,21***	0,22***	0,86***	1
(12) SES	0,21***	0,24***	0,25***	0,10***	0,18***	0,21***	0,15***	0,17***	0,28***	0,29***	0,27***

SES, socio-economic status. Sensitivity, maternal sensitivity in mother–child interaction. Stimulation, maternal stimulation in mother–child interaction. Picture book, frequency of joint picture book reading. w, wave. *p < 0,05, **p < 0,01, ***p < 0,001.

### SES-Related Disparities in HLE Process Measures: Mixed-Effects Regression Models

Table 3 shows the results of the three separate mixed-effects regression models on SES-related disparities in mother’s sensitive interaction behavior, mother’s stimulating behavior, and the frequency of joint book reading. Using a combined SES measure of maternal education, HISEI, and household income, SES was significantly related to all three HLE process characteristics; these effects range between 0,12 and 0,25. For maternal sensitivity, the SES effect did not change significantly across waves. Yet, as far as mother’s stimulating behavior is concerned, the effect of SES changed across waves. For joint picture book reading, again a changing effect across waves was found, particularly in wave three compared to the first assessment wave.

**TABLE 3:** Coefficients for the three mixed-effects linear regression models.

	**Sensitivity**	**Stimulation**	**Picture book**
SES	0,25***	0,12***	0,18***
**Wave (ref wave1)**			
Wave 2	−0,03	−0,01	−0,01
Wave 3	−0,01	−0,002	0
**SES × wave (ref wave 1)**			
SES × wave 2	0,05	0,11*	0,03
SES × wave 3	0,05	0,12**	0,16***
Intercept	−0,01	−0,01	0
**Random-effects parameters**			
*Sd* (intercept)	0,36	0,51	0,51
*Sd* (residual)	0,91	0,85	0,83

Coefficients presented in SD units. SES, socio-economic status. Sensitivity, maternal sensitivity in mother–child interaction. Stimulation, maternal stimulation in mother–child interaction. Picture book, frequency of joint picture book reading. Ref, reference group. *p < 0,05, **p < 0,01, ***p < 0,001.

### Predicting Child Language at Age Two: Structural Equation Modeling

Using structural equation modeling we analyzed the effects of structural (SES) and the more proximal HLE process characteristics of the home learning environment on the children’s vocabulary and grammar at age two, first separately for each wave and then conjointly for all three waves.

#### Disparities in Early Vocabulary: Effects of the Families’ SES and HLE Process Characteristics

**FIGURE 1:**
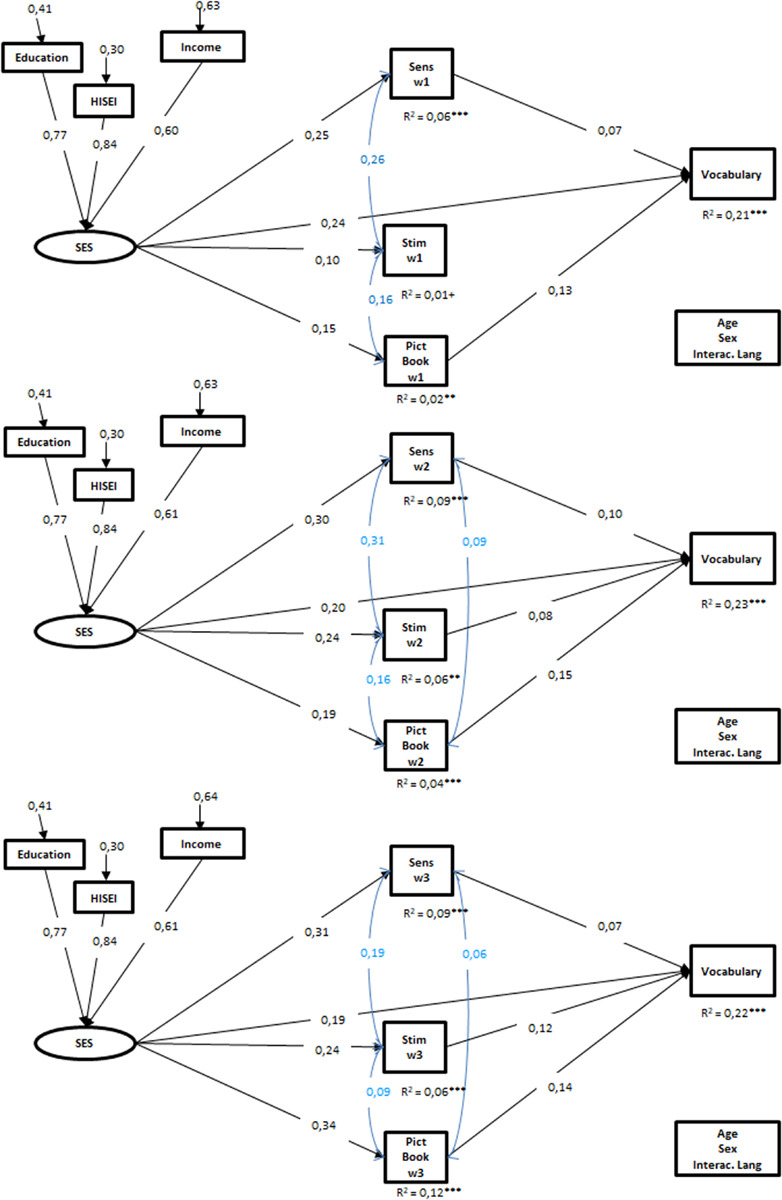
Results of the structural equation models for vocabulary, separated for each wave. Only the significant standardized coefficients are presented (*p* < 0,05). *N* = 2.272; SES, socio-economic background; Sens, maternal sensitivity in mother–child interaction; Stim, maternal stimulation in mother–child interaction; Pict Book, frequency of joint picture book reading; w, wave. Model1: CFI = 0,98, RMSEA = 0,03, SRMR = 0,02, Model2: CFI = 0,98, RMSEA = 0,03, SRMR = 0,02, Model3: CFI = 0,98, RMSEA = 0,03, SRMR = 0,02. ^+^*p* < 0,10, *p < 0,05, **p < 0,01, ***p < 0,001.

**FIGURE 2:**
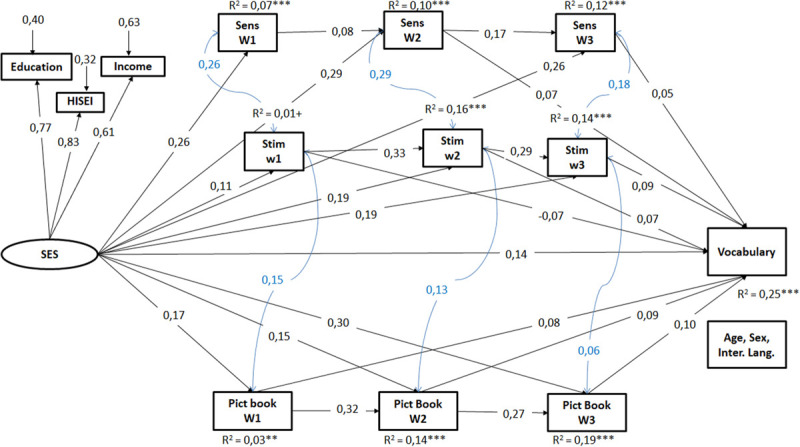
Results of the structural equation model for vocabulary, model above all three waves. Only the significant standardized coefficients are presented (*p* < 0,05). SES, socio-economic background; Sens, maternal sensitivity in mother–child interaction; Stim, maternal stimulation in mother–child interaction; Pict Book, frequency of joint picture book reading; w, wave. *N* = 2.272; CFI = 0,96, RMSEA = 0,03, SRMR = 0,04. ^+^*p* < 0,10, *p < 0,05, **p < 0,01, ***p < 0,001.

All three separate models as well as the integrated model demonstrated sufficient fit to the data (see Figures 1, 2). In all four SEMs, the latent construct SES significantly predicted the vocabulary of the children at age two.

In the separate models, families’ SES showed a direct path to all three HLE characteristics. Further, mother’s sensitivity and the frequency of joint picture book reading at first, second, and third wave predicted child vocabulary at age two. Hence, children with comparatively more sensitive mothers, and parents who often engaged in joint picture book reading showed a more advanced vocabulary compared to children with less sensitive mothers and parents who reported less joint picture book reading. Further, mother’s stimulating behavior in wave 2 and 3 predicted the children’s vocabulary at 2 years of age but not in wave 1 when children were 7 months of age. Together, SES and the HLE process characteristics explained about 21% (wave 1), 23% (wave 2), and 22% (wave 3) of the variance in children’s vocabulary.

The integrated model including the HLE process predictors from all three assessment waves substantiates and extends the results of the separate models. First, families’ SES proved to be directly associated with mother’s sensitivity and stimulation behavior as well as with the frequency of joint picture book reading at each wave, even when considering all waves at the same time. Furthermore, we found a direct effect of SES on children’s vocabulary at age two despite considering the three process characteristics across waves in the model. Second, differences in the frequency of joint picture book reading were moderately stable across waves with each wave showing a direct effect on child vocabulary at age two. Third, the sensitivity of the mother (i.e., her prompt and responsive behavior in mother–child interaction) in wave 2 and 3 also predicted child vocabulary positively while maternal sensitivity in the first year of life did not. Stability of maternal sensitivity in mother–child interactions across waves was rather low. Fourth, the stimulation behavior of the mother in the first wave was negatively associated with child vocabulary while it was increasingly positively associated in wave 2 and 3 with a moderate stability across waves.

Overall, SES and all predictors in the full model explained only slightly more variance in children’s vocabulary (25%) than the separate models.

#### Disparities in Early Child Grammar: Effects of the Families’ SES and HLE Process Characteristics

**FIGURE 3:**
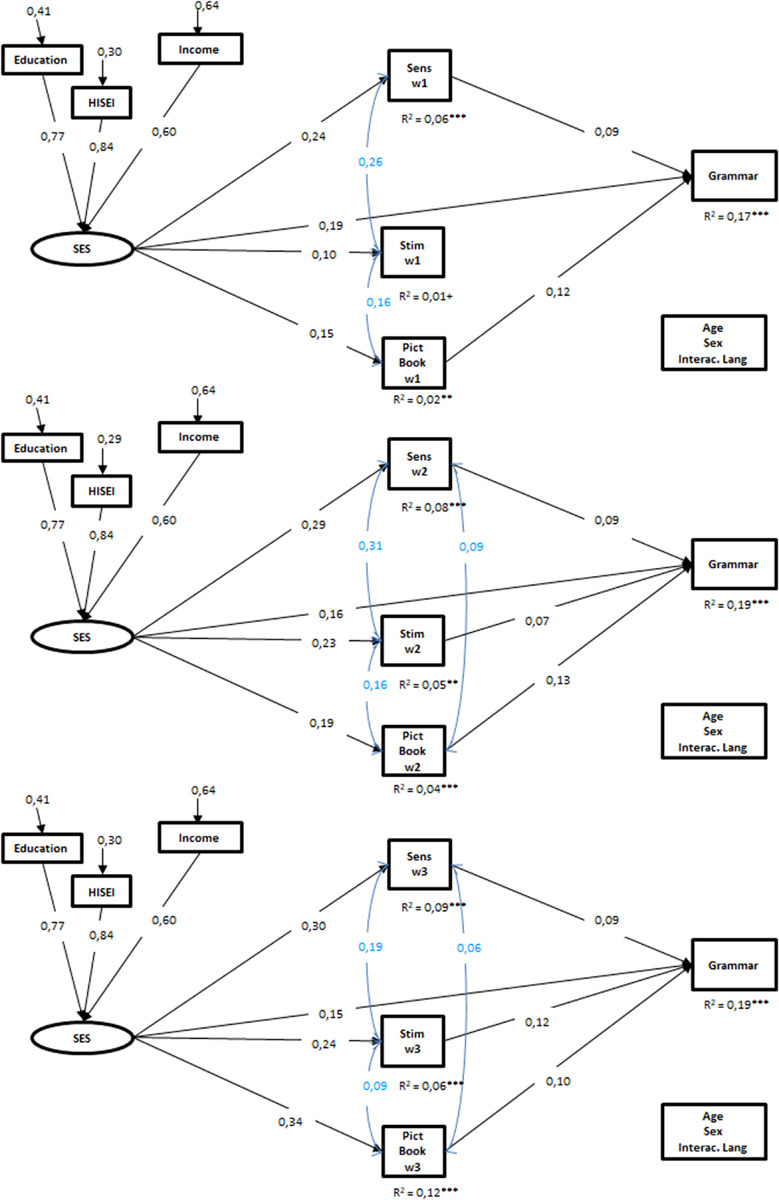
Results of the structural equation models for Grammar, separated for each wave. Only the significant standardized coefficients are presented (*p* < 0,05). SES, socio-economic background; Sens, maternal sensitivity in mother–child interaction; Stim, maternal stimulation in mother–child interaction; Pict Book, frequency of joint picture book reading; w, wave. *N* = 2.272; Model1: CFI = 0,98, RMSEA = 0,03, SRMR = 0,02, Model2: CFI = 0,98, RMSEA = 0,03, SRMR = 0,02, Model3: CFI = 0,98, RMSEA = 0,03, SRMR = 0,02. ^+^*p* < 0,10, *p < 0,05, **p < 0,01, ***p < 0,001.

**FIGURE 4:**
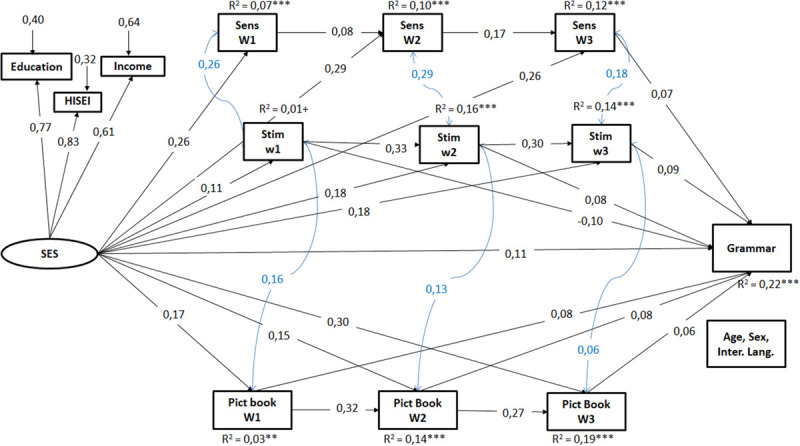
Results of the structural equation model for grammar, model above all three waves. Only the significant standardized coefficients were presented (*p* < 0,05). SES, socio-economic background; Sens, maternal sensitivity in mother–child interaction; Stim, maternal stimulation in mother–child interaction; Pict Book, frequency of joint picture book reading; w, wave. *N* = 2.272; CFI = 0,96, RMSEA = 0,03, SRMR = 0,03. ^+^*p* < 0,10, *p < 0,05, **p < 0,01, ***p < 0,001.

Focusing on child grammar, the results were similar to those reported for early vocabulary. All four models showed sufficient fit to the data (see Figures 3, 4). In all four models, the latent SES construct directly predicted child grammar at the age of 2 years in each of the models.

Further, in the three separate models, the latent SES construct also predicted each of the HLE process characteristics and, in each wave, the three process characteristics were positively associated with the grammatical skills of the children with the exception of maternal stimulation behavior in wave 1. SES and the process characteristics explained 17% (wave 1) and 19% (wave 2 and 3) of the variance in the grammatical skills of the children.

The integrated model including SES and the HLE process indicators from all three waves as predictors also shows a comparable picture for early child grammar and vocabulary. First, the latent SES construct predicted each of the HLE process characteristics at each wave even when considering all waves simultaneously as well as the grammar skills of the children at the age of two. Second, for each wave, the model shows a direct effect of the frequency of joint picture book reading on the grammatical skills of the children at age two. Third, the stimulation behavior of the mother in the first wave was negatively associated with early grammar outcomes, while later on (wave 2 and 3), the grammar skills of children were increasingly positively associated with the stimulating behavior of the mothers. Fourth, concerning mother’s sensitivity in mother–child interaction, we did not find direct effects of the early waves; there was only a positive effect of the third wave on the children’s grammar skills. Overall, SES and HLE process predictors from three waves explained 22% of the variance of the children’s grammar skills at age two.

Table 4 presents an overview of the results concerning early vocabulary and grammar outcomes and the predictive impact of the various predictors across waves highlighting the similarity of the pattern of results for both language components.

**TABLE 4:** Relation of SES, HLE process characteristics, and child language – direct effects within the integrated models including all waves.

	**Vocabulary**	**Vocabulary**	**Vocabulary**	**Vocabulary**	**Grammar**	**Grammar**	**Grammar**	**Grammar**
SES	0,24***	0,20***	0,19***	0,15***	0,19***	0,16***	0,15***	0,11***
Sensitivity w1	0,07**			0,04	0,09**			0,05+
Sensitivity w2		0,10**		0,07*		0,09*		0,06
Sensitivity w3			0,07**	0,05*			0,09***	0,08**
Stimulation w1	−0,02			−0,07**	−0,04+			−0,10**
Stimulation w2		0,08*		0,07*		0,07*		0,08*
Stimulation w3			0,12***	0,09***			0,12***	0,09***
Picture book w1	0,13***			0,08***	0,12***			0,08***
Picture book w2		0,15***		0,09***		0,13***		0,08***
Picture book w3			0,14***	0,10***			0,10***	0,06*
Age	0,18***	0,19***	0,18***	0,19***	0,23***	0,23***	0,23***	0,23***
Sex	0,14***	0,13***	0,13***	0,13***	0,14***	0,13***	0,13***	0,13***
Interaction language	−0,18***	−0,17***	−0,16***	−0,16***	−0,12***	−0,12***	−0,11***	−0,11***

*R*^2^	0,21***	0,23***	0,22***	0,25***	0,17***	0,19***	0,19***	0,22***

Coefficients presented in SD units. SES, socio-economic background; Sensitivity, maternal sensitivity in mother–child interaction. Stimulation, maternal stimulation in mother–child interaction. Picture book, frequency of joint picture book reading. W, wave. Ref, reference group. ^+^p < 0,10, *p < 0,05, **p < 0,01, ***p < 0,001.

## Discussion

In line with theoretical assumptions as outlined in educational frameworks of the home learning environment (e.g., Kluczniok et al., 2013) as well as empirical results, SES-related disparities in language development have been suggested to be mainly transferred by differences in process characteristics of the home learning environment which are themselves influenced by the families’ socio-economic status. There is no doubt that the family is the most important learning environment in the first years for most children. Yet so far, only a few studies focused on the dynamics of parenting behavior across the first years (Lugo-Gil and Tamis-LeMonda, 2008; Rodriguez and Tamis-LeMonda, 2011; Vallotton et al., 2017) and their potentially changing effects on the language development of children. Hence, the aim of the present study was to take a longitudinal perspective on three process characteristics of the home learning environment and their association with SES as well as with two aspects of children’s language development, namely vocabulary and grammar. These two subdomains of language development have been suggested to be differentially related to differences in the home learning environment (Vasilyeva and Waterfall, 2011).

In particular, the present paper addressed the following main research issues and questions: First, extending the findings on SES-related disparities in the three HLE process characteristics across the first 2 years of children’s lives as well as their potentially changing associations across these 2 years. Second, investigating the predictive effects of socio-economic family background and the longitudinally assessed HLE process characteristics on vocabulary size and on early child grammar. These analyses also addressed the question of whether the relation between SES and HLE process characteristics as well as their predictive association with child language differ across assessment waves. Drawing on attachment theory, on Vygotsky’s concepts of social learning in the zone of proximal development (Vygotsky, 1978), and on literacy research, we focused on (a) maternal sensitivity (sensitive responsiveness) to child signals, (b) mother’s cognitively stimulating behavior, and (c) the frequency of joint picture book reading which all have been suggested to foster child development from early on.

Our results show that the families’ socio-economic background is associated with all three HLE process characteristics – maternal responsive sensitivity and stimulating interaction behavior in mother–child interaction as well as the frequency of joint picture book reading – at each of the three assessment waves conducted during the first 2 years of the children’s life. Thus, mothers with lower SES interacted with their child less sensitively and in ways that were less stimulating than mothers with a higher SES. And parents with lower SES also engaged less often in joint picture book reading with their child. Hence, we replicated previous research results which also showed an association between the socio-economic status of the family and different HLE process characteristics (e.g., NICHD Early Child Care Research Network, 1999; Bradley et al., 2001; Farrant and Zubrick, 2012; Lehrl et al., 2012). Using also the Newborn Cohort Study of the NEPS, Attig and Weinert (2018) already documented an association between the quality of maternal interaction behavior in the second year of life and the education level of the mother. Further, not only maternal interaction behavior has been shown to be related to the families’ SES but also – for example – joint activities of the parents with their child such as the frequency and quality of joint book reading (Niklas and Schneider, 2010; Farrant and Zubrick, 2012; Lehrl et al., 2012). The present study extends these results and shows their associations across time (by including three measurement points) and indicators, e.g., by using a combined measure of SES and by differentiating between maternal sensitivity and stimulation behavior which are often combined into a global measure of the quality of mother’s interaction behavior (e.g., Weinert et al., 2017). Even more importantly, we investigated the effects of the families’ SES on the three HLE process measures longitudinally and found that the SES effect on maternal sensitivity stayed stable across the first 2 years, whereas the SES effect on mother’s stimulation behavior as well as on the frequency of joint picture book reading changed across the waves. Thus, the associations between SES and the latter two process measures seem to get stronger across the first 2 years of the children’s lives hinting to the importance of early intervention.

Turning to the second research question, our results showed that across the three measurement points nearly all process characteristics of the HLE predicted the children’s vocabulary and grammar skills at the age of two. Yet, the explained variation in the joined model including all measurement points is not really higher than in the separate models. Again, the results replicate previous research which also showed an association between different process characteristics of the home learning environment and the children’s language development (Bus et al., 1995; Tamis-LeMonda et al., 1996, 1998; NICHD Early Child Care Research Network, 1998; Bromley, 2009; Nozadi et al., 2013). For example, Vallotton et al. (2017) found that the importance of early maternal sensitivity and stimulating interaction behavior for later child vocabulary changes across the first 3 years of life. Whereas at the age of 14 months, maternal sensitivity seems to have a greater impact on vocabulary, the effect changed until the age of 36 months with maternal stimulation having a greater effect on vocabulary compared to sensitivity. In the present study, we also see changes across the assessment waves with differences in maternal stimulation behavior at the age of 17 and 26 months predicting the language skills of the children, whereas the effect at the age of 7 months was not significant (in the separate models) or even negative (in the global model). In contrast, maternal sensitivity in mother–child interaction predicted the language skills of the children in the separate models in each wave, while in the global model – considering all waves simultaneously – a significant direct effect only appeared for wave 2 and 3 for vocabulary and wave 3 for grammar, but not for the earlier wave(s). Yet, when comparing the effects of maternal sensitivity and stimulating behavior across waves, in line with the results of Vallotton et al. (2017), in the first waves it is particularly maternal sensitivity that seems to foster children’s language development while mother’s stimulation behavior seems to become comparatively and increasingly more important in the later waves.

However, it is important to note that parental sensitivity and stimulation behavior is often defined and coded in different ways and may thus cover partially different concepts across studies. For example, in the present paper we defined maternal sensitivity as a prompt, sensitive, and adequate responsivity to the child’s behavior and signals in mother–child interaction and mother’s stimulation behavior as going beyond the child’s actual level of action and development thus fostering child development by providing the child with new aspects, materials, and suggestions for exploration (see Linberg A. et al., 2019). Other conceptualizations focus on domain-specific maternal parenting behavior, differentiating, for example, socio-emotional supportive parenting behavior (which includes responsivity, sensitivity, and positive regard with a particular focus on socio-emotional signals of the child) and cognitive-verbally stimulating interaction behavior (see Linberg, 2018; Linberg et al., 2020). Further, differentiating sensitive and prompt maternal responsivity from mother’s scaffolding and child-adapted stimulating behavior as well as differentiating socio-emotional supportive behavior from cognitive-verbally stimulating behavior may be difficult when using global measures; more detailed coding may help to address these differentiations and their (differential) impact on child behavior and development more in depth (Linberg, 2018). Yet as our measures of maternal sensitivity and stimulating behavior are not highly correlated and as their relative impact seems to change over time, the pattern of results seems to support the assumption that they cover different process characteristics, with sensitivity being particularly relevant in the very early phases of child development and a growing impact of stimulating behavior over time. When interpreting these results, it should also be considered that the sensitivity and the stimulation measure at the age of 26 months and the children’s language skills in the present study were measured at the same time point.

Overall, variation in parent behavior (see for example for maternal responsiveness, Bornstein et al., 2008) seems effective as parents adapt their behavior to the developmental status of their child (Rodriguez and Tamis-LeMonda, 2011). As Bowlby (1988) suggested, a prompt, sensitive, and adequate response to the child’s signals may help the child to gain a feeling of competence and being valued. Further, this might be particularly relevant in the very early phases of development as the mother focuses on the child’s needs, his/her attentional focus and interests, and this may help the child to learn to regulate his/her behavior and to understand the very first words and communicative function of language based on a common ground of the interactional exchange. Later on, adaptive stimulation that goes beyond the child’s actual action might become more important as the child starts to follow these hints, offers, and suggestions more actively (Baldwin, 1995).

Not surprisingly, joint picture book reading in each wave predicted the later language skills of the children at the age of 2 years. Thus, our results extend the findings of Bromley (2009) who showed that picture book reading at the age of 10 months was associated with the language skills of children at the age of 34 months. Our data add to this finding that already at the age of 7 months picture book reading is associated with later language development and that the effect still remains even when considering later joint picture book reading as well as other characteristics of the HLE within the same model. The results are in line with research showing a relation of joint reading with language development not only at this young age (e.g., Bromley, 2009) but also for preschoolers (Bus et al., 1995; Sénéchal et al., 2008). This latter research also highlights the importance of the quality of joint picture book reading as well as specific differential associations with different language measures such as vocabulary, grammar, or early literacy (Lehrl et al., 2012; Anderka, 2018). Unfortunately, the NEPS data does not include measures of the quality of joint picture book reading and related questions should be investigated in further studies for even younger children as the specific impact of interactional characteristics during joint picture book reading, i.e., which characteristics are particularly important in promoting language development, seem to change with development.

Besides the HLE process characteristics, the families’ socio-economic status also proved to be related to child vocabulary and grammar skills at age two. This converges with previous findings from the Newborn Cohort Study of the NEPS which showed an effect of education on the vocabulary (Linberg et al., 2020) and on a language measure which takes vocabulary and grammar into account (Attig and Weinert, 2019). Further, the results are in line with other studies which also showed an association between language and the families’ socio-economic background at the age of 2 years (Fernald et al., 2013; Law et al., 2019). Weinert and Ebert (2013) found that the social background measured with a combined SES construct accounted for 15,6% of the differences in the language skills of 4-year-old children. An increase of SES-related disparities over time was shown in studies focusing on even older children (Linberg and Wenz, 2017; Volodina et al., 2020). A mediation of the SES effect on child language through the process characteristics was not directly investigated in the present paper. Yet, although the structural equation models considered all process characteristics and, in the joint models all process characteristics across the three waves, together with the effects of SES on the process measures, there was still a direct effect from the SES to both measures of child language. This result hints to the assumption that the parenting behavior considered did not (fully) mediate and thus cannot (fully) explain the SES effect on the children’s language skills. Linberg et al. (2020) showed with the same data set from the NEPS that early language-stimulating interaction behavior only mediated 9% of the effect of maternal education on vocabulary development in the second year of life. It is up to future research to investigate which (other) mechanisms could explain the effect of SES on early child language. Further, even in the two global models that included HLE characteristics of three measurement points as well as family SES and some control variables, the models only explained 25% and 22% of the variation of the children’s language skills. Hence, other child characteristics as well as facets of the home learning environment, for example more domain-specific aspects such as maternal guiding language (Shruti et al., 2018), may also influence language development.

Adding to previous research, the present study focused not only on one aspect of language development but took vocabulary size as well as grammar skills of the children into account. It has been assumed that in the early stages grammar is less influenced by the learning environments compared to vocabulary (Anderka, 2018 for a brief overview; see also the results of Huttenlocher et al., 2010). In line with social-cognitive theories of language acquisition (Tomasello, 2003; Weinert and Grimm, 2018) and extending the results of Weinert and Ebert (2013) to even younger children, the present results showed that nearly all process characteristics as well as the families’ socio-economic background predicted vocabulary size and grammar skills at the age of two to about the same extent. Hence, both aspects of language development are influenced by the home learning environment in the first years of life. Note, however, that our grammar indicator was rather superficial as it drew on a parent report measure (see e.g., Newport et al., 1977 for more sophisticated measures). Further, we used rather unspecific characteristics of the home learning environment. In fact, drawing on other studies, we suspect different facets of the home learning environment to affect vocabulary and grammar development differently at least beyond age three, as it has been shown that these subdomains of language development are differentially related to different process characteristics of the home learning environment (Lehrl et al., 2012; Anderka, 2018) which also explain their relation to families’ SES (Anderka, 2018).

### Strength and Limitations

First of all, using data from a large longitudinal cohort study is one of the strengths of the current study. Second, different process characteristics including observational measures as well as a comprehensive indicator of the families’ socio-economic background were considered in joint models. Third, two subdomains of language development, namely vocabulary and grammar, were analyzed allowing to differentiate the effects for these two aspects of language development.

Besides several strengths of the study, the study also has important limitations. First, as a language measure we used a standardized parental report measure (ELFRA; Grimm and Doil, 2006). Of course, a potential bias, related to the social status of the parents, in answering the questionnaire can’t be ruled out, and hence a misjudgment of the results should be considered. However, the ELFRA is a well-established instrument which has been shown to significantly correlate with language test scores (Sachse et al., 2007). Further, it is not unusual to work with such checklists (see for example Nozadi et al., 2013; Morgan et al., 2015; Law et al., 2019) especially in large panel studies because testing children at the age of two by standardized tests appears to be difficult in large-scale assessments (Weinert et al., 2016). Further, our results concerning the association between SES and early language skills are in line with previous research (e.g., Fernald et al., 2013; Law et al., 2019) supporting their validity. Fortunately, at a later age, the NEPS applied a standardized language test (Peabody Picture Vocabulary test, Dunn and Dunn, 2007; Lenhard et al., 2015) so that for older children a potential bias can be excluded. Second, due to the split design in the second wave which randomly assigned only half of the sample to take part in the observational assessments, the study had to handle a high amount of missing data. Although the dropouts mainly resulted from the split design, this should be considered when interpreting the results. Further research should underpin the current results to make the interpretation and conclusions stronger. As a third limitation, the measurement of the three HLE process characteristics should be mentioned. Thus, the interactional measures were derived from a 5 (wave 1) or 10 min (wave 2 and 3) interaction situation with standardized material. Although control studies showed some stability of maternal interaction behavior, this situation is rather short and thus may underestimate differences between mothers’ behavior. In addition, the measures of all three HLE process characteristics considered are based on a single rating scale each. In fact, when aggregating across scales, the stabilities of the quality of maternal interaction behavior is much higher (see e.g., Freund et al., 2019). Without doubt, to use more scales or more differentiated codings would be desirable to make the constructs and also the results more robust. Further, although the paper addresses three different process characteristics of the home learning environment, only positive parenting and interaction behavior that was not domain-specific has been considered. Further research may not only differentiate between sensitivity and stimulation behavior but may also include, for example, domain-specific language stimulation behavior (see Linberg et al., 2020 for an example).

## Conclusion

Taking the families’ socio-economic status as well as various process characteristics of the home learning environment, such as different characteristics of maternal interaction behavior and the frequency of joint picture book reading across the first 2 years into account, will help to better understand what happens in families in the first 2 years and what precisely impacts the language development of children. First, as assumed, significant associations were found between the socio-economic family background and all three HLE process characteristics, with two of them showing a change in their association across the waves. Second, our results clearly show that not just one aspect of parent behavior is associated with the children’s language development, but all three aspects are related to child language with at least partially changing effects across early child development. Further, the direct effect of the socio-economic background remained even after including the HLE process characteristics from all three measurement points. In addition, across waves the various aspects did not just exert influence via the same measure at a later time-point but most measures also asserted a direct effect from earlier waves. Interestingly, the present results showed that in the first 2 years, and with respect to the rather domain-general aspects of the HLE characteristics considered, a comparable effect on vocabulary and grammar was demonstrated. This is in contrast to research with older children that showed different facets of the home learning environment to be differentially related to vocabulary and grammar acquisition (Lehrl et al., 2012; Anderka, 2018).

## Data Availability Statement

The present article analyzed data of the National Educational Panel Study in Germany. The anonymized data is available for the scientific community at http://www.neps-data.de.

## Ethics Statement

The NEPS study is conducted under the supervision of the German Federal Commissioner for Data Protection and Freedom of Information (BfDI) and in coordination with the German Standing Conference of the Ministers of Education and Cultural Affairs (KMK) and – in the case of surveys at schools – the Educational Ministries of the respective Federal States. All data collection procedures, instruments and documents were checked by the data protection unit of the Leibniz Institute for Educational Trajectories (LIfBi). The necessary steps are taken to protect participants’ confidentiality according to national and international regulations of data security. Participation in the NEPS study is voluntary and based on the informed consent of participants. This consent to participate in the NEPS study can be revoked at any time. All parent of the Newborn Cohort of the NEPS give their agreement for participation and answering questions during the assessments as well as a written consent for participating in the video-taped measures to each measurement point.

## Author Contributions

MA and SW contributed to the conception and the design of the manuscript. MA performed the statistical analysis and wrote the first draft of the manuscript. SW revised the manuscript critically to improve the draft and contributed to the Theoretical Background and Discussion sections. Both authors contribute to manuscript revision, read and approved the submitted version.

## Conflict of Interest

The authors declare that the research was conducted in the absence of any commercial or financial relationships that could be construed as a potential conflict of interest.

## Footnotes

1 This paper uses data from the National Educational Panel Study (NEPS): Starting Cohort Newborns, *doi: 10.5157/NEPS:SC1:6.0.0*. From 2008 to 2013, NEPS data was collected as part of the Framework Program for the Promotion of Empirical Educational Research funded by the German Federal Ministry of Education and Research (BMBF). As of 2014, NEPS is carried out by the Leibniz Institute for Educational Trajectories (LIfBi) at the University of Bamberg in cooperation with a nationwide network.

2 In most of the families, the mother was the primary caregiver. Hence, only a few fathers took part in the interviews (wave 1: 22, wave 2: 8, wave 3: 49). These cases were handled as missing for the parent–child interaction to reduce gender specific variance (Mills-Koonce et al., 2015; see Linberg A. et al., 2019 for a similar approach).
